# Alcohol Induces Sensitization to Gluten in Genetically Susceptible Individuals: A Case Control Study

**DOI:** 10.1371/journal.pone.0077638

**Published:** 2013-10-15

**Authors:** Stuart Currie, Nigel Hoggard, Matthew J. R. Clark, David S. Sanders, Iain D. Wilkinson, Paul D. Griffiths, Marios Hadjivassiliou

**Affiliations:** 1 Academic Unit of Radiology, University of Sheffield, Sheffield, United Kingdom; 2 Department of Gastroenterology, Sheffield Teaching Hospitals NHS Foundation Trust, Sheffield, United Kingdom; 3 Department of Neurology, Sheffield Teaching Hospitals NHS Foundation Trust, Sheffield, United Kingdom; UCSF, United States of America

## Abstract

**Background:**

The mechanisms of cerebellar degeneration attributed to prolonged and excessive alcohol intake remain unclear. Additional or even alternative causes of cerebellar degeneration are often overlooked in suspected cases of alcohol-related ataxia. The objectives of this study were two fold: (1) to investigate the prevalence of gluten-related serological markers in patients with alcohol-related ataxia and; (2) to compare the pattern of brain involvement on magnetic resonance imaging between patients with alcohol and gluten ataxias.

**Materials & Methods:**

Patients diagnosed with alcohol and gluten ataxias were identified from a retrospective review of patients attending a tertiary clinic. HLA genotype and serological markers of gluten-related disorders were recorded. Cerebellar volumetry, MR spectroscopy and voxel-based morphometric analyses were performed on patients and compared with matched control data.

**Results:**

Of 904 registered patients, 104 had alcohol ataxia and 159 had gluten ataxia. 61% of the alcohol ataxia group and 70% of the gluten ataxia group had HLA DQ2/DQ8 genotype compared to 30% in healthy local blood donors. 44% of patients with alcohol ataxia had antigliadin antibodies compared to 12% in the healthy local population and 10% in patients with genetically confirmed ataxias. None of the patients with alcohol ataxia and antigliadin antibodies had celiac disease compared to 40% in patients with gluten ataxia. The pattern of structural brain abnormality in patients with alcohol ataxia who had antigliadin antibodies differed from gluten ataxia and was identical to that of alcohol ataxia.

**Conclusions:**

Alcohol related cerebellar degeneration may, in genetically susceptible individuals, induce sensitization to gluten. Such sensitization may result from a primary cerebellar insult, but a more systemic effect is also possible. The duration and amount of exposure to alcohol may not be the only factors responsible for the cerebellar insult.

## Introduction

Previous studies have shown that patients with chronic alcohol abuse often have elevated serological levels of antibodies directed towards self-antigens as well as elevated IgA immunoglobulins and T-cells[1]. High levels of immunoglobulins can be seen in immune mediated diseases[2]. Recently, excessive alcohol consumption has been shown to mediate an IgA immune response, which is not only directed towards alcohol-derived neo-antigens but also against tissue transglutaminase[3]. Transglutaminases constitute a family of enzymes with cross-linking capability[4]. Tissue transglutaminase 2 (TG2) and TG3 have been implicated as the autoantigens in celiac disease[5] and dermatitis herpetiformis respectively[6]. Recently, antibodies against TG6 were found in patients with gluten ataxia (GA – defined as idiopathic sporadic ataxia with positive antigliadin antibodies)[7]. Individuals with GA (and other gluten-related disorders) show genetic susceptibility, with almost all patients demonstrating the HLA-DQ2/DQ8 genotype[8,9]. 

The presence of TG2 antibodies (the autoantigen of celiac disease) in patients with chronic alcoholism raises the possibility of alcohol-induced sensitivity to gluten. One potential mechanism recently proposed is that alcohol-induced intestinal mucosal lesions increase gut permeability and may lead to the exposure of new antigens, (such as gliadin peptides), which are considered foreign by the mucosal system[3]. A compromise to the blood brain barrier (such as is thought to occur in gluten ataxia[10] and alcohol abuse[11]) could theoretically, expose the brain to antibodies or immune complexes and lead to/potentiate neurological damage. 

Both gluten-related diseases and alcohol are known to affect the cerebellum, an organ that shows particular vulnerability to immune-mediated damage. Indeed, a number of conditions exist that are associated with immune-provoked cerebellar damage such as, paraneoplastic cerebellar degeneration, post-infectious cerebellitis, Miller-Fisher syndrome, opsoclonus-myoclonus ataxia and ataxia with anti-GAD antibodies[12]. Many of these conditions are associated with autoantibodies that target and react with Purkinje cells causing their loss and permanent disability (ataxia) for the patient[12,13]. Recent evidence also suggests the internalization of circulating immunoglobulins by Purkinje cells in the setting of blood brain barrier disruption[14,15]. 

Given that gluten exposure (in cases with GA) and alcohol are known to cause cerebellar degeneration, it may be difficult to establish the primary cause of the cerebellar insult in any patient that demonstrates co-existence of the two conditions. 

The primary aim of this study was to investigate the prevalence of serological evidence of sensitivity to gluten and HLA-status in patients with ataxia presumed to be due to chronic alcohol abuse (ACAA). The secondary aim was to compare the pattern of cerebellar involvement using magnetic resonance (MR) imaging between patients with GA and patients with ACAA (with and without serological evidence of sensitivity to gluten). 

## Materials & Methods

### Subjects and Controls

The study was approved by the local, regional ethics committee (Leeds Central) and all participants gave their written informed consent prior to inclusion in compliance with the Code of Ethical Principles for Medical Research Involving Human Subjects of the World Medical Association (Declaration of Helsinki). 

A retrospective review of all patients attending the ataxia clinic, Royal Hallamshire Hospital, Sheffield, UK was performed. Patients with a diagnosis of GA and ACAA were sought. This clinic was established 15 years ago and cares for over 900 patients with progressive ataxia. Patients are reviewed on a regular basis and have undergone extensive investigations to establish the cause of their ataxia. A diagnostic breakdown of these patients is available in the literature[16] but briefly consists of both familial (183/900) and sporadic (717/900) ataxia groups. Patients with familial ataxia included episodic ataxia (types 1 and 2), spinocerebellar ataxia (types 1, 2, 3, 6, 7, 8, 13, 15, 17, 28), Friedreich’s ataxia, Niemann-Pick disease, familial British dementia, mitochondrial encephalopathy lactic acidosis and stroke-like episodes, Gerstmann-Staussler-Scheinker syndrome and polymerase gamma. Patients with sporadic ataxia largely consisted of idiopathic sporadic ataxia, gluten ataxia, alcoholic cerebellar degeneration, clinically probable multiple system atrophy, paraneoplastic ataxia, and drug-induced ataxia. GA is defined as an idiopathic sporadic ataxia with positive antigliadin antibodies[17]. A diagnosis of ACAA was based upon positive clinical history of chronic alcohol excess with hematological/serological evidence of alcohol excess (raised gamma-GT and mean corpuscular volume) in the absence of an alternative explanation for the ataxia. All patients from the two groups that had undergone the exact same MR imaging protocol of the brain (see below) were selected. The clinical notes of these patients were then assessed to obtain the duration and severity of ataxia symptoms at the time of MR imaging. Ataxia severity was classified as mild, moderate or severe as assessed by the same experienced consultant neurologist (MH) and adapted from previously published data[18]. Briefly the scale comprised: mild – instability without staggering steps or falls; moderate – instability with staggering steps or falls and/or requires support through a walking aid and; severe – unable to walk despite support from an accompanying person. 

HLA-DQ2/DQ8 serological status of both groups was obtained, as was the presence of antigliadin antibodies in the ACAA group at the time of MR. Evidence of celiac disease (in the form of positive duodenal biopsies) was also sought in both patient groups. The presence of antigliadin antibodies allowed subdivision of the ACAA group in to those with (ACAA+) and those without (ACAA-) serological evidence of gluten sensitivity.

Healthy volunteers, recruited for research, were selected for age- and sex-matching from a database of 55 subjects that had undergone the same MR imaging protocol as detailed below. Subject details have been extensively reported in the literature[19]. 

### MR Imaging Outcome Measures

Outcome measures comprised of: (a) cerebellar volume, expressed as a percentage of total intracranial volume (%CV:TIV); (b) spectroscopic N-acetyl aspartate to creatine (NAA/Cr) and Choline to creatine (Cho/Cr) ratios in the cerebellar vermis and hemisphere and; (c) grey matter ‘density’ as indicated by grey matter voxel-based morphometry. Data from each subject group were compared to control data matched for age and sex.

### Imaging Protocol

Scanning was performed at the Academic Unit of Radiology, University of Sheffield using a 3-T system (Philips ACHIEVA 3.0T Best, Netherlands) with an 8-channel receive only array head coil. 

Structural MR imaging comprised high-resolution 3-dimensional T1-weighted MR imaging datasets acquired using a magnetization-prepared rapid gradient echo sequence (TR = 11 ms, TE = 4.8 ms, TI= 1400 ms, flip angle = 8°, volume-of-view = 256 x 205 x 150 mm^3^, voxel dimension = 0.8 mm isotropic, acquisition time = 5 min 32 sec). 

**Figure 1:**
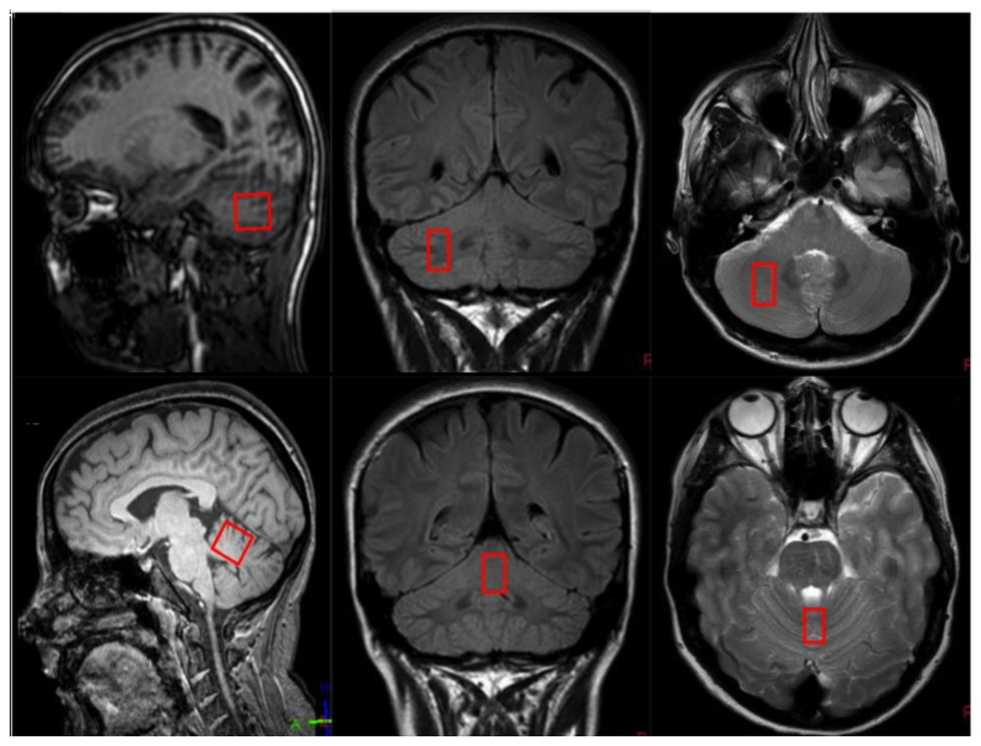
Cerebellar voxel placement. Location of ^1^H MR spectroscopy voxel within the right cerebellar hemisphere (top row) and superior vermis (bottom row).

^1^H-MR spectroscopy comprised a point-resolved spectroscopy (PRESS) sequence (TR = 2000 ms, TE = 144 ms; 128 measurements; 1024 spectral points; spectral bandwidth 2000Hz) with data acquired at two voxel positions. Each voxel comprised a parallelepid of 2.0 X 1.0 X 2.0 cm^3^ (4 mL) placed over the superior cerebellar vermis and the deep cerebellar white matter of the right cerebellar hemisphere. Care was taken to avoid inclusion of cerebrospinal fluid spaces within the volume of interest. (Figure 1) After automated higher-order shimming, water suppression was achieved using a chemical shift selective imaging pulse technique (CHESS). Post-processing of the spectra involved the following steps: zero filling, gaussian filtering, exponential multiplication, Fourier transform and manual phase correction with baseline subtraction. Fitted-peak integral values for N-acetylaspartate (NAA) and choline (Cho) were referred to the peak integral value of creatine (Cr) as the internal reference using the MR system manufacturer’s proprietary software. NAA/Cho data was also assessed.

Decision regarding the quality of cerebellar spectroscopy was made by consensus opinion between two neuroradiologists (NH/SC) and was based on criteria published previously in the literature[20]. Briefly, this comprised assessment of signal to noise ratio, peak shape and separation of choline (Cho) and creatine (Cr) peaks. Patients with poor quality cerebellar MR spectra were excluded.

### Structural Imaging Analysis

#### a): Whole brain and cerebellar volume analysis

Brain tissue volume, normalized for subject head size, was estimated with Structural Image Evaluation, using Normalization, of Atrophy cross-sectional method (SIENAX)[21,22], part of FSL[23–27]. 

Cerebellar segmentation was obtained in the following manner using FSL. Firstly, a cerebellum segmentation template was obtained from a standard template image using an Integrated Registration and Segmentation Tool (FIRST)[28]. The template image was then registered using FMRIB’s Non-linear Image Registration Tool (FNIRT). Invwarp was used to invert the FNIRT warp and transfer the segmentation from the template space to the subject’s space. For all subjects and controls cerebellar volume (CV) (calculated from binarised data of the segmented cerebellum) was expressed as a ratio of total intracranial volume (TIV) to allow for variation in individual head size.

#### b): Grey matter voxel-based morphometry

Structural data was analyzed with FSL-VBM, a voxel-based morphometry style analysis[29,30] carried out with FSL tools[23]. The following method has been previously published in the literature[31]. First, structural images were brain-extracted using BET[24]. Next, tissue-type segmentation was carried out using FAST4[27]. The resulting grey-matter partial volume images were then aligned to MNI152 standard space using the affine registration tool FLIRT[25,26], followed by nonlinear registration using FNIRT, which uses a b-spline representation of the registration warp field[32]. The resulting images were averaged to create a study-specific template, to which the native grey matter images were then non-linearly re-registered. The registered partial volume images were then modulated (to correct for local expansion or contraction) by dividing by the Jacobian of the warp field. Finally the modulated segmented images were then smoothed with an isotropic gaussian kernel having a sigma of 3 mm[33]. 

### Statistical Analysis

Any difference in mean %CV:TIV between subjects and controls was assessed using an Independent-Samples Mann-Whitney U Test (SPSS software (SPSS, Inc.)). This method was also applied to examine any statistical differences in mean NAA/Cr and Cho/Cr ratios of the cerebellar hemisphere and vermis between subjects and controls. Bonferroni correction was applied to allow for multiple comparisons. Age- and sex-matched control groups were obtained for both GA and ACAA cohorts. To investigate grey matter density changes, we used permutation-based non-parametric inference within the framework of the general linear model (5000 permutations) through FSL[34]. Results were considered significant for P<0.05, fully corrected for multiple comparisons (i.e. automatically controlling for family-wise error rate)[34].

## Results

Over a 15-year period, 904 patients with progressive cerebellar ataxia attended the specialist ataxia clinic at the Royal Hallamshire Hospital, Sheffield, UK. Of these, 104 (11.5%) patients were diagnosed with ACAA and 159 (17.6%) patients with GA.

61% of the ACAA group and 70% of the GA group had the HLA DQ2/DQ8 genotype, compared to 30% in healthy local blood donors[35]. 44% of patients with ACAA had antibodies to gliadin (IgG and/or IgA) compared to 12% in the healthy local population and 10% in patients with genetically confirmed ataxias[36,37]. None of the patients with ACAA and antigliadin antibodies had celiac disease on duodenal biopsy compared to 40% of patients with GA who had enteropathy on biopsy. None of the patients with GA had raised MCV or gamma GT. 

Twenty-nine patients with ACAA and 17 patients with GA underwent the previously detailed, same MR imaging protocol. Table 1

**Table 1:** Patient demographic data according to subject group*.

Subject group	Mean age (SD and range) at MRI (yrs.)	M:F	Median duration of symptoms at MRI (yrs.)	Mean ataxia severity score (1=mild, 2=moderate, 3=severe)
GA (n=17)	52±10 (33-67)	5:12	10 (range 3-16)	1.9
ACAA (n=29)	54±8 (39-69)	17:12	6 (range 1-10)	1.7
ACAA+ (n=10)	49±8 (39-62)	5:5	5 (range 1-10)	1.7
ACAA- (n=19)	56±8 (41-69)	12:7	8 (range 2-10)	1.7

* Controls underwent the exact same MR imaging protocol as the patient groups. Controls were matched to patients within each group according to age and gender from a pool of 55 healthy volunteers. Control demographics matched to the GA group: n = 17, M:F = 5:12, mean age at MRI = 52±10 (33 to 68) and; for the ACAA group: n = 29, M:F = 17:12, mean age at MRI = 55±8 (41 to 68). No statistically significant differences in age were found across the ACAA, GA and control groups (p = 0.74, Kruskal-Wallis test).

### MR Spectroscopy

#### Patients with ACAA vs. controls

Vermian NAA/Cr was significantly reduced in patients with ACAA (0.86 ± 0.11) compared to age- and sex-matched controls (0.97 ± 0.05), CI 95% 0.05 to 0.16; p <0.001. No statistically significant difference was found between patients with ACAA and controls for vermian Cho/Cr (p=0.73) and cerebellar hemispheric NAA/Cr (p=0.19) and Cho/Cr (p=0.37). 

#### Patients with GA vs. controls

Vermian NAA/Cr was significantly reduced in patients with GA (0.77 ± 0.11) compared to age- and sex-matched controls (0.96 ± 0.07), CI 95% 0.12 to 0.25; p <0.001. No statistically significant difference was found between patients with GA and controls for vermian Cho/Cr (p=0.13) and cerebellar hemispheric NAA/Cr (p=0.63) and Cho/Cr (p=0.39). 

#### Patients with GA vs. patients with ACAA

Using group data as depicted in Table 1 (i.e. unmatched for symptom duration), vermian NAA/Cr was significantly reduced in patients with GA (0.77 ± 0.11) compared to patients with ACAA (0.86 ± 0.11), CI 95% 0.02 to 0.16; p <0.05. When patients were matched for symptom duration (GA n=11, ACAA n=17; median 8 years from symptom onset to MR; mean age 52±7 years for both groups; mean ataxia severity GA=1.6, AA=1.5) there was an overall trend for reduced vermian NAA/Cr in the GA group compared to ACAA but this failed to reach statistical significance (0.79 vs. 0.87 respectively, CI 95% -0.19 to 0.03, p>0.05). 

No statistically significant differences were found in vermian Cho/Cr (p=0.10/p=0.20) or cerebellar hemispheric NAA/Cr (p=0.30/p=0.23) and Cho/Cr (p=0.64/p=0.78) between patients with GA and patients with ACAA irrespective if the data were unmatched or matched for symptom duration (p values shown = unmatched/matched data).

Comparing MR spectroscopy data from GA with ACAA- and GA with ACAA+ revealed no statistical significant differences in neurometabolite ratios at either voxel position irrespective of matching for symptom duration. Similarly, no statistically significant differences were found in the neurometabolite ratios when ACAA- and ACAA+ groups were compared directly, irrespective of voxel position and matching for symptom duration. 

### Cerebellar Volume

#### Patients with ACAA vs. controls

Cerebellar volume (as a percentage of total intracranial volume) was significantly smaller in patients with ACAA (5.95 ± 0.79) compared to age- and sex-matched controls (8.85 ± 1.11); CI 95% 2.26 to 3.53, p<0.001.

#### Patients with GA vs. controls

Cerebellar volume was significantly smaller in patients with GA (4.71 ± 1.20) compared to age- and sex-matched controls (8.46 ± 1.06); CI 95% 2.96 to 4.54, p<0.001.

#### Patients with GA vs. patients with ACAA

Cerebellar volume was significantly smaller in patients with GA (4.71 ± 1.20) compared to patients with ACAA (5.95 ± 0.79); CI 95% 0.57 to 1.93, p<0.001. Statistical significance was maintained when patients were matched for duration of symptoms (GA n=11; ACAA n=17; %CV:TIV = 4.90 ± 1.28 vs. 5.83 ± 0.78 respectively; CI 95% 0.13 to 1.72, p<0.05 - median duration of symptoms 8 years; mean ataxia severity score GA 1.6, ACAA 1.5, mean age both 52 ± 7 years). 

Cerebellar volume was significantly smaller in patients with GA compared to patients with ACAA- (4.71 ± 1.20 vs. 5.60 ± 1.64; CI 95% 0.49 to 1.90, p<0.05). Although the trend for smaller cerebellar volume in the GA group (compared to ACAA-) continued when patients were matched for duration of symptoms, data failed to reach statistical significance (p = 0.07). Similarly, when cerebellar volume in patients with GA was compared with that of ACAA+ no statistical significance was demonstrated irrespective of matching for symptom duration (p = 0.19/0.62; unmatched/matched for symptom duration). 

No statistically significant difference in cerebellar volume was found between ACAA+ and ACAA- irrespective of matching for symptom duration (p = 0.11/0.39; unmatched/matched for symptom duration). 

### Grey Matter VBM

**Figure 2:**
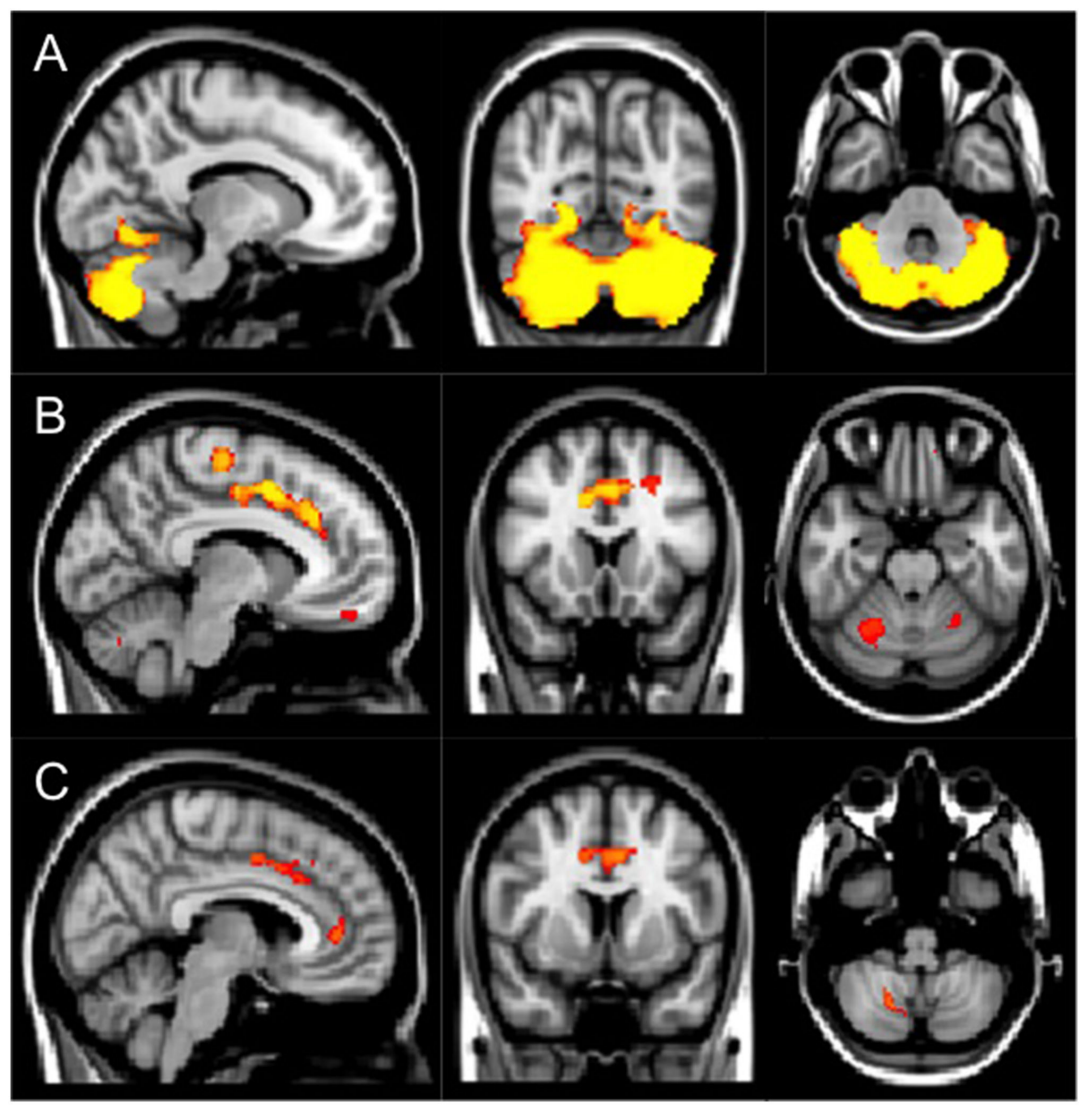
Grey matter voxel-based morphometry. Areas of reduced grey matter density (depicted on the MNI152 brain) compared to age- and sex-matched controls using voxel based morphometry (highlighted areas, corrected, p<0.05). A) Patients with GA show pancerebellar grey matter reduction. B) Patients with ACAA show cerebellar hemispheric grey matter reduction as well as cingulate gyrus and inferior frontal lobe involvement. C) Patients with ataxia due to chronic alcohol abuse that also demonstrate sensitivity to gluten. Note this is the same pattern of disease as shown in patients with ACAA (B) and differs to that of GA(A).

Voxel-based morphometry demonstrated reduced grey matter volume in both patient groups compared with controls. This constituted pure cerebellar atrophy in the GA group but cerebellar atrophy in combination with supratentorial involvement in the ACAA group. Analysis of just the ACAA+ group compared with controls showed a similar pattern of atrophy to that of the ACAA group. Figure 2

## Discussion

The primary aim of this study was to investigate the prevalence of serological evidence of sensitivity to gluten and HLA-status in patients with ataxia presumed to be due to chronic alcohol abuse (ACAA). The secondary aim was to compare the pattern of cerebellar involvement using MR imaging between patients with GA and patients with ACAA (with and without serological evidence of sensitivity to gluten). We specifically targeted the superior cerebellar vermis on MR spectroscopy as this is a site known to be affected by both diseases[38,39]. Until now, a measurement of the prevalence of gluten sensitivity in patients with ACAA was unavailable in the medical literature. Additionally, we know of no previous reports in the literature that have quantified *in vivo* cerebellar volume and that have performed voxel based morphometry to characterize grey matter loss in both patient cohorts. 

This study demonstrates that the prevalence of antigliadin antibodies in patients with ACAA is significantly higher than in control groups (44% vs. 12% in the healthy population[36] and 10% in hereditary ataxias[39]). Furthermore, the prevalence of the HLA-DQ2/DQ8 genotype in patients with ACAA is more than double that of the healthy population (61% vs. 30%[35]). This study also demonstrates that compared to age- and sex-matched controls, patients with ACAA and GA have significant structural and functional cerebellar deficits. NAA is considered a marker of neuronal health and integrity. Accordingly, reduced NAA/Cr levels are typically attributed to neuronal dysfunction or loss. Our MR spectroscopy findings are in agreement with the published literature[40–42]. 

Voxel-based morphometry analysis reveals that GA and ACAA have different radiological phenotype with respect to grey matter loss. Patients with GA show considerable grey matter loss, which appears to be confined to and involves most of the cerebellum. Conversely, patients with ACAA have more modest cerebellar involvement but the disease extends into the supratentorial compartment. Our finding of reduced grey matter density in the anterior cingulate gyrus in patients with ACAA is in agreement with reports of regional volume loss in alcohol dependents and has been linked to dysfunction in the brain reward system in those individuals[43]. 

One particular question arising from this study concerns the origin of antigliadin antibodies in patients with ACAA. The high prevalence of antigliadin antibodies together with the high prevalence of HLA-DQ2/DQ8 suggests that this is more than just an epiphenomenon. The differing pattern of brain abnormality between patients with GA and ACAA on voxel based morphometry suggests that in the case of ACAA, antigliadin antibodies may arise after a cerebellar insult rather than being the cause of it *per se*. It also implies that there is a genetic susceptibility (HLA DQ2) for patients with ACAA, something that has not previously been reported. The HLA DQ2 has also been seen in a number of other autoimmune diseases including primary autoimmune cerebellar ataxia. This raises the issue that autoimmunity may have a role to play in the development of ataxia in patients that drink excessively.

The lack of histological evidence of celiac disease in patients with ACAA despite positive antigliadin antibodies as compared to the 40% prevalence of celiac disease in patients with gluten ataxia (all positive for antigliadin antibodies) appears to provide an argument against the gastrointestinal tract being the primary source of the immunological response. A possible explanation may be that antigliadin antibodies arise in patients with genetic susceptibility (HLADQ2/DQ8 genotype) following a chronic immunological insult (alcohol) centered on the cerebellum. Alcohol has previously been associated with antibodies to transglutaminase and is known to evoke an immunogenic reaction[3]. 

The role of tissue specific transglutaminases (i.e. TG2 in celiac disease, TG3 in dermatitis herpetiformis and TG6 in GA) in ACAA remains to be explored. TG6 is a primarily brain expressed TG and antibodies to TG6 have been found in the serum and in the cerebellum of patients with GA[7]. 

This study is limited by the relatively small sample size. It is also retrospective and as such has inherent limitations. Antigliadin antibodies were used as evidence for sensitivity to gluten simply because currently there are no sensitive serological markers for the whole spectrum of gluten-related disorders. We have not examined whether patients had co-existing alcoholic liver disease and also have not compared the data with a cohort of patients with alcoholic liver disease without ataxia. As such we have not discounted the possibility that liver disease *per se* may be a risk factor for serological positivity for gluten-related antibodies rather than being mediated through a prolonged cerebellar insult. Kril and Butterworth looked at formalin-fixed sections of the anterior superior aspect of the cerebellar vermis in 36 patients (30 alcoholic and 6 nonalcoholic) with autopsy-proven cirrhosis and found cerebellar degeneration in 20 cases (17 alcoholic and 3 nonalcoholic patients). The degree of Purkinje cell loss and gliosis was graded as mild in all 3 nonalcoholic patients (compared to 8 mild, 6 moderate and 3 severe for the alcoholic group). The authors raised the possibility that liver disease-related mechanisms may contribute to cerebellar pathology but this is still to be confirmed[44]. Floreani et al. examined the prevalence of antigliadin antibodies in 67 patients with chronic liver disease of different aetiology, including 29 with primary biliary cirrhosis, 31 with chronic non-A non-B hepatitis, and 7 with autoimmune chronic active hepatitis. Overall, low prevalence of IgA antigliadin antibodies was observed in the three patient groups (3.4% primary biliary cirrhosis, 3.2% non-A, non-B hepatitis compared to 1.3% of patients with inflammatory bowel disease). One patient with autoimmune chronic active hepatitis had raised IgA and IgG antigliadin antibodies and subsequent jejunal biopsy confirmed celiac disease. Not all patients had small bowel biopsy and no neurological correlation was investigated[45]. Germenis and colleagues retrospectively assessed the prevalence of tissue transglutaminase (IgA tTGAbs) in 738 patients with chronic liver diseases and compared data to that from 1,350 healthy controls. The overall prevalence of tTGAb in the patient group was 6.4% - significantly higher compared to 0.3% of healthy controls. Aetiology of liver disease was proven not to be a significant risk factor for the detection of tTGAbs (prevalence of tTGAb in patients with alcoholic liver disease 7.8%, nonalcoholic fatty liver disease 8.2%, chronic viral hepatitis 4.3% and autoimmune hepatitis 12%). Only 2 patients were diagnosed with celiac disease compared to 4 in the healthy controls. The authors concluded that IgA anti-tTG reactivity in patients with chronic liver diseases is not associated with celiac disease but appears to be associated with the presence of an autoimmune phenomena and cirrhosis. However, only 29 patients (of the 46 that tested positive for tTGAbs) underwent small bowel biopsy and celiac disease was confirmed in 2 (all 4 healthy controls with raised tTGAbs had biopsy confirmed celiac disease). HLA typing was also limited. Additionally, the presence of neurological dysfunction was not examined[46].

Future prospective studies examining the prevalence of serological evidence (including TG6) of gluten-related disorders in a cohort of patients with alcoholic liver disease would be of interest as abnormal liver function tests and autoimmune hepatitis are over-represented in patients with celiac disease. Research examining cerebrospinal fluid for any evidence of intrathecal antibodies to transglutaminases as well as evidence of oligoclonal bands and compromise to the blood brain barrier in these patients would also be of interest. 

In summary, this study demonstrates that patients with alcoholic ataxia have a high prevalence of antigliadin antibodies in combination with a high prevalence of the HLA-DQ2/DQ8 genotype, strongly associated with gluten-related disorders. The pattern of cerebellar involvement in alcoholic ataxia differs from that observed in patients with GA. This raises the possibility that alcohol excess in genetically susceptible individuals may induce sensitization to gluten rather than these being cases of GA. 
